# The Influence of Ethnic and Mainstream Cultures on African Americans’ Health Behaviors: A Qualitative Study

**DOI:** 10.3390/bs7030049

**Published:** 2017-08-04

**Authors:** Ewelina M. Swierad, Lenny R. Vartanian, Marlee King

**Affiliations:** 1 School of Psychology, UNSW Sydney, Sydney, NSW 2052, Australia; e.swierad@psy.unsw.edu.au; 2 School of Social Sciences and Psychology, University of Western Sydney, Penrith 2751, Australia; Marlee.King@westernsydney.edu.au

**Keywords:** culture, health, ethnic culture, mainstream culture, health behaviors, picking and choosing, healthy makeovers

## Abstract

Background: Culture plays an important role in shaping individuals’ health behaviors. This qualitative research examines the relationship between African Americans’ ethnic and mainstream cultures and their health behaviors (i.e., food intake and physical activity). Methods: This study used in-depth semi-structured interview format with a group of 25 African Americans to examine the influence of ethnic and mainstream culture on African Americans’ food intake and physical activity. Thematic analysis was used to identify common themes and patterns related to African Americans’ health behaviors as well as to report these patterns within data. Results: The present study found that African Americans position both their ethnic and mainstream culture as important influences on their health behaviors pertaining to food intake and physical activity. Most participants reported taking advantage of “the best of both worlds” by engaging in *picking and choosing* healthy behaviors from both cultures to which they belong, and they perceived preparing *healthy makeovers* as a way to optimize their health. They also identified a range of practical considerations that can facilitate or hinder engagement in healthy eating and physical activity (e.g., affordability, social support). Participants discussed a number of other positive (e.g., resilience, spirituality) and negative (e.g., experience of discrimination) influences on health behaviors. Conclusions: African Americans consider both their ethnic and mainstream cultures important in shaping their health behaviors. These cultural influences need to be understood in the context of other psycho-socio-environmental factors that affect individuals’ health behaviors. The current study has practical implications for designing health promotion programs for African Americans.

## 1. Introduction

African Americans and members of other minority groups are disproportionately affected by overweight and obesity [1,2,3,4]. Recent data suggest that, although obesity rates in the US have stabilized in the general population, rates of obesity continue to increase among minority groups, specifically non-Hispanic black women and Mexican American women [3]. Obesity rates are highest among African Americans, affecting 45.0% of non-Hispanic Blacks, as compared to 36.8% of Mexican-Americans, and 30.6% of Whites [5]. Half of African American women and 37% of African American men are obese [6]. African Americans also have one of the lowest rates of physical activity among all ethnic groups in the US [7,8] and are also 38% less likely to meet recommendations for fruit and vegetable consumption as compared to White Americans [9]. Understanding the factors that contribute to these disparities in health and health behaviors is an important part of developing effective preventions. (Note that, in this paper, we use the term “health behaviors” to refer to the broad range of behaviors related to food intake and physical activity.)

It is widely recognized that culture can shape people’s food intake and physical activity [10,11,12] because culture represents set of values, social norms, traditions, and ways of doing things and engaging with the world that are transmitted across generations and influence behaviors [13]. In particular, culture shapes health related values, norms, beliefs, and behaviors through people’s connection to their social and physical environments [14]. Given that individuals’ social and physical environments change, the influence of culture and culture itself can change as well. Thus, it is also important to recognize that culture embodies not only fixed but also fluid qualities [13]. Some aspects of culture are fixed (e.g., the collectivistic or individualistic focus of a given culture) in that they are transmitted over generations, but other aspects are fluid in that they evolve as the social environment becomes increasingly diverse (e.g., culturally preferred foods that evolve as immigrants come and bring their “own culture” to an existing culture) [15].

Most research in the area of culture and health has focused exclusively on the influence of individuals’ ethnic culture on their health behaviors [16,17,18]. Here we consider ethnic culture to reflect a group of individuals of the same ethnic background sharing similar values, norms, and engaging in similar practices or behaviors. For instance, Airhihenbuwa et al. [16] explored African Americans’ perceptions about positive and negative cultural food practices and the contexts in which they occur. Their participants reported that, not only does ethnic culture affect individuals’ food choices, but culture also influences how the food is prepared and the context in which it is consumed by creating social norms surrounding these practices.

In addition to exploring the influence of individuals’ ethnic culture, the health practices of African Americans also need to be also considered in the context of their mainstream culture (i.e., the most popularized or typical beliefs, norms, and practices—including health related trends surrounding food intake and physical activity—common within American culture). Because African Americans belong to both their ethnic and mainstream cultures, they are exposed to two sets of cultural norms surrounding food intake and physical activity. These multiple cultural norms can potentially have positive or negative influences on individuals’ health behaviors. The negative influence is related to the idea that norms of different cultures can be in conflict with each other. Given that cultural norms affect health behaviors, bicultural groups, such African Americans, may be simultaneously exposed to discrepant cultural lifestyle-related norms, and these norms may adversely affect their food intake and physical activity. A few previous qualitative studies explored the relationship between cultural norm discrepancy and health behaviors [18,19,20,21]. For example, in one of these qualitative studies, participants reported feeling conflicted between culturally informed eating habits, body size image, and mainstream culture dietary guidelines [21]. Such conflicting norms can contribute to poor health behaviors [11]. For example, they may confuse individuals with regard to what constitutes healthy lifestyle and make it more difficult for them to actually follow any of the norms promoting healthy behaviors that each culture has to offer. Furthermore, the confusion caused by conflicting norms might itself trigger inaction. The exact processes involved in cultural norm conflict and health behaviors, however, are yet to be identified.

There is also a potential positive influence of ethnic and mainstream cultures on one’s health, and this is related to the idea that bicultural individuals may have more access to, and more knowledge about, health behaviors that are valued in each of these cultures and that shape their health practices. To date, however, there is very little research examining the ways in which African Americans perceive positive aspects of both cultures influencing health and the ways in which they can capitalize on health enhancing qualities of each culture. Thus, it is important to expand the exploration of culture on health and health behaviors by examining the simultaneous influence of African Americans’ ethnic and mainstream cultures, particularly in terms of the positive factors contributing to better health.

The idea that belonging to two different cultures can positively influence one’s actions, such as health behaviors, is supported by research on Bicultural Identity Integration [16,22]. According to this construct, exposure to two cultures provides people with an opportunity to learn and benefit from their rich cultural environment [17,23]. Embracing both parts of one’s culture can enhance people’s lives [18,19,24,25]. For instance, bicultural identity integration was found to increase individuals’ creativity [20,26] and their wellbeing [18,19,24,25]. In the context of health, exposure to two cultures affords individuals two sources of information about health trends and practices common in each culture, and these different sources of influence can shape individuals’ own health behaviors. For example, one can be exposed to behaviors common in mainstream American culture such as the trend toward healthy eating, and behaviors common in ethnic African American culture such as consuming foods at home with one’s family. As a result, individuals can have a broader range of healthy behaviors to choose from while pursuing their healthy lifestyle. These potential benefits of being exposed to two cultures highlights the value in examining the simultaneous influence of African Americans’ ethnic and mainstream culture on their health behaviors.

Of course, the influence of culture on individuals’ health behaviors cannot be separated from their immediate social context and broader physical environment [12]. For example, research has shown that, because African Americans value collectivism and togetherness, family and friends play an important role in shaping their health behaviors [27,28]. Furthermore, the health behaviors of African Americans are often the result of health and economic disparities among this cultural group. African Americans (and many other minority groups) are disproportionately more likely than White Americans to experience factors such as compromised access to healthy food [29,30], limited access to safe places for physical activity [31] and low affordability of healthy foods [32], and these factors can impact their likelihood of engaging in health behaviors. Thus, the health behaviors of African Americans need to be examined in the context of personal factors, individuals’ immediate social environment, and their physical environment [33,34].

### The Present Study

There are a few gaps in the previous research on culture and health that the current study attempted to address. First, previous studies have focused on the relationship between culture and African Americans’ health behaviors only in the context of African Americans’ ethnic culture [11,16]. These studies did not take into account the simultaneous influence of individuals’ mainstream and ethnic cultures on their health practices. Given that African Americans are simultaneously exposed to both their ethnic and mainstream American cultures, the present study examined the role of both cultures in individuals’ food intake and physical activity.

Second, there are relatively few studies directly examining the interplay between culture and other psycho-socio-environmental factors that collectively affect individuals’ health practices [11,16,18]. Because the opportunities to engage in healthy behaviors among African Americans depend on the myriad of interconnected personal, social, and environmental factors, the present study explored the influence of culture on African Americans’ food intake and physical activity in the context of these factors. Adopting a qualitative methodological approach can help develop a richer understanding of the role of culture and other psychological, social, and environmental factors in African Americans’ health behaviors [11,16,17,35].

Third, most recent studies have focused exclusively on unhealthy norms related to food intake and physical activity, omitting the healthy aspects of individuals’ cultures. This approach represents a “deficit model,” which, according to previous research, can be counterproductive in health promotion programs [36]. Therefore, in addition to the examination of health compromising (or unhealthy) factors connected to individuals’ cultures, the present study focused predominantly on healthy behaviors from both cultures that may optimize African Americans’ health and explored how receptive African Americans are to the idea of *picking and choosing* healthy behaviors from their ethnic and mainstream cultures as a means of optimizing their health.

The specific goals of the present qualitative study were to examine: (1) what is the role of people’s ethnic and mainstream culture in shaping their health behaviors; (2) how do individuals perceive the idea of *picking and choosing* healthy behaviors from their ethnic and mainstream culture to optimize their health; and (3) what psycho-socio-environmental factors optimize the positive influence of culture on African Americans’ food intake and physical activity?

## 2. Methods

### 2.1. Research Design

This study used an in-depth semi-structured interview format to examine the influence of ethnic and mainstream cultures on African Americans’ health behaviors. This approach was selected because qualitative research has been considered effective in exploring cross-cultural issues related to health, including food intake and physical activity [11,16,17,35]. Semi-structured interviews allow participants reflect freely on their own experiences and observations [37] in their own words rather than categorizing and quantifying of experiences of research participants on pre-established quantitative scales [38]. The interview format enables researchers to obtain detailed insight about participants’ experiences, perceptions, beliefs, values, and behaviors [37], which can be particularly important in examining concepts that are relatively unexplored, such as the role of individuals’ ethnic and mainstream cultures in their health behaviors.

### 2.2. Participants and Recruitment Strategies

Participants were 25 individuals recruited from Columbia University and from Harlem, New York, USA (a neighborhood near the university that is largely African American) to participate in individual interviews about “healthy lifestyle related behaviors”. Study advertisement fliers were posted throughout the Columbia University campus, and also in churches and grocery stores in Harlem. To be eligible for the study, participants needed to self-identify as African American. Participants were paid USD $20 for their participation in the study.

The majority of the sample (18 participants) was recruited from Columbia University. The remaining seven participants were recruited from Harlem, and they were not associated with Columbia University. See Table 1 for detailed demographic characteristics of the sample. With regard to participants’ cultural identification, participants were asked a single question about whether they identified more with their ethnic or mainstream culture. There was variability in the extent to which participants identified with their ethnic vs. mainstream culture (see Table 1).

The results section of the study will refer to particular participants’ responses designated by a participant number. See Appendix A for information on each participant’s level of education and cultural identification.

### 2.3. Procedure

Commencing each interview, the researcher introduced herself and explained the broad purpose of the study. After providing informed consent and permission to audio record the interview, participants completed a brief questionnaire assessing their gender, level of education, and cultural self-identification. The researcher then read the interview questions in sequence, implementing a semi-structured interview format. This format involved using pre-determined probes, such as brief open-ended questions or clarification statements to help participants elaborate on some important themes. Interviews were held over a three-week period at Columbia University. The interview questions aimed to explore the cross-section of culture and health, and focused specifically on two research questions: (1) “culture, barriers to, and facilitators of, healthy lifestyle among African Americans”; and (2) “the simultaneous influence of both one’s ethnic and mainstream cultures on African Americans’ health behaviors”. Interview questions (see Table 2) were developed based on a combination of complementary strategies which included: (1) literature review of qualitative studies related to health behaviors among African Americans [11,16,18]; (2) conversations with a four self-identified members of African American culture; and (3) consultation with an African American researcher in the field of psychology to ensure the cultural sensitivity of the questions. The interviews lasted on average 40 min.

### 2.4. Ethical Considerations

The study was approved by UNSW ethics committee, and the permission to recruit participants for the study was obtained from the Institutional Review Board at Columbia University.

### 2.5. Thematic Analysis

The interviews were transcribed by three independent transcribers and de-identified. Each participant was assigned a number (e.g., “Participant 1”). Interview transcripts were then imported into NVivo version 10 (QSR International, Melbourne, Australia) [39], a qualitative data analysis computer software package.

Thematic analysis [37] was used to identify common themes and patterns related to African Americans’ health behaviors as well as to report these patterns within data. Thematic analysis recognizes language as a tool for storytelling that reflects people’s broader social and physical environments [40]. In other words, this approach highlights the ways in which people make sense of their experiences that are informed by their social and environmental context [41]. Understanding the context of people’s experiences, such as culture and the psycho-socio-environmental factors examined in this study, seems to be particularly important in the case of members of minority groups (i.e., African Americans in the context of this study) that are often discriminated against and overpowered by external, systemic forces affecting their lives [40]. Therefore, this method is well-suited to examine the perceptions and experiences of African Americans with regards to their health behaviors.

Following the thematic analysis guidelines created by Braun and Clarke [37], the first step was ‘in-depth familiarization’ of data by reading transcripts multiple times. This enabled the generation of initial codes, which involved organizing data into meaningful groups and identifying potentially important information within a data set. Codes were then organized into themes. This process started from analyzing the patterns between previously identified codes and considering how different codes may connect with each other forming an overarching theme [37]. Once the final thematic framework was established, the names of the themes were further refined so that they fit the story that the data were illustrating. During each stage of analysis, the analyses were initially conducted by the first author and were then discussed in detail with the second and third authors. This ensured that there was a consistency within patterns identified, and that the analysis was plausible.

## 3. Results

Two broad themes were identified throughout the interviews: (Section 3.1) Culturally-derived barriers to and facilitators of a healthy lifestyle and (Section 3.2) Practical considerations beyond culture when adopting a healthier lifestyle.

Within the first primary theme, (Section 3.1) *Culturally-derived barriers to and facilitators of a healthy lifestyle*, a number of subthemes were identified, including: (Section 3.1.1) “It’s a strength”: Drawing upon ethnic culture to improve health behavior; (Section 3.1.2) Difficulties changing a culturally ingrained proclivity for “unhealthy” behaviors; (Section 3.1.3) The “healthy” individual: Aligning health beliefs and behaviors with that of mainstream culture; (Section 3.1.4) “The best of both worlds”: Combining cultural and mainstream approaches to health; and (Section 3.1.5) The importance of support from the ethnic and mainstream community.

Within the second theme, (Section 3.2) *Practical considerations beyond culture when adopting a healthier lifestyle*, the responses were coded under the following subthemes: (Section 3.2.1) “It’s is not worth it to eat healthy”: The cost of having a healthy lifestyle; (Section 3.2.2) Access to healthy foods, healthy living and education, and (Section 3.2.3) Personal factors and motivation.

### 3.1. Culturally-Derived Barriers to and Facilitators of a Healthy Lifestyle

Within this theme, African Americans described how their ethnic culture and their mainstream culture was a source of strength and a source of weakness to their health behaviors, detailing culturally-derived barriers to and facilitators of a healthy lifestyle.

#### 3.1.1. “It’s a Strength”: Drawing upon Ethnic Culture to Improve Health Behavior

When asked to describe what cultural values, norms, or beliefs help African Americans lead a healthy lifestyle, many people spoke of resilience as a culturally valued trait that historically has helped African Americans to deal with slavery, oppression, and discrimination. This ability to endure externally imposed pain and discomfort in order to improve one’s life circumstances was identified as a beneficial characteristic that African Americans can apply to healthy lifestyle. For example, *Participant 19* described the importance of resilience in facilitating health behaviors saying:

“I feel like, our strengths and determination have help with healthy lifestyle; we tend to be very determined in doing anything we want to do; and it it’s a fight; it’s a strength and a fight to stand through a lot of harsh stuff. I feel like that strength, if it can be just implemented into healthy living, then I think that determination to want to change lifestyle from eating the traditional, unhealthy, greasy, heavily salted, to healthy living can change a lot of things in the culture”.

Evident in the excerpt above, *Participant 19* attributes African Americans’ experience with adversity and their determination as a strength that assists them when faced with challenges such as changing a culturally embedded practice—eating some traditional unhealthy foods. This idea of collective resilience emphasizes the notion of strength and pride associated with African American values.

In the same vein, several participants indicated that resilience, or as *Participant 11* puts it “you can do-itness”, can be a powerful source of healthy behaviors, helping people to explore creative ways of eating healthy and exercising. That is, resilience helps people overcome some of the barriers to healthy living common within African American communities. For example, *Participant 13* emphasized the importance of resilience by saying:

“In terms of the strengths, I think the strengths we have is that we’re a resilient people and we know that we have the potential within us to improve, but we just need… we need to make it a culture of speaking and engaging with each other about the things that are relevant to our health and happiness. And our eating habits. And once we can spread that word we can make that become more of a culture in the black community in terms of what we eat. I think it will make a tremendous difference. There are traces of that starting already”.

Evident in the above excerpt is the notion that resilience goes beyond strength and becomes more of an issue of awareness, connected to happiness and health. The concept of resilience was also apparent in *Participant 16’s* account:

“With the little that we some of us, many of us do have we’ve learnt to like stretch and make a lot of it. Even with like the food that we have a lot of our ethnic food came from like. Even back from when we were enslaved and we like, like we had like you know not the best portions of particular animal or, um but we learnt how to you know like make it really good”.

The importance of church or spirituality was also discussed as a factor that helps people engage in healthy eating and physical activity. Many participants talked about “spiritual connection” or “religion” as essential support systems that facilitate healthy lifestyle. *Participant 6* explained the role of spirituality in enhancing African Americans health behaviors and talked about the ways in which church can help in promoting healthy living:

“In my church they encourage you to eat healthy and if you’re stressed out, church is an avenue where you can find some type of relief so I would say that church would be one of the biggest things that helps”.

Similarly, *Participant 5* spoke about a church as a place that provides a sense of community in which people work together on common goals and inspire one another to lead better and healthier lives: “If you’re part of a church and your church group goes walking every day you’re more likely to go walking with a group so you could talk and socialize and work out”.

According to some participants, spirituality may also provide guidelines for the way people should treat their bodies. *Participant 4* emphasized her belief that, “your body is your temple from God and you’re supposed to take care of it.” She further stated:

“This is the body that God gave me and I need to make sure I’m doing the best I can with it to; taking care of myself honors God because you know, gluttony is a sin and you’re not supposed to over-eat, you’re not supposed to do all of this stuff. So it’s the spirituality and the appreciation of the body and the understanding what when I eat healthy, I feel better”.

Noteworthy is how *Participant 4* emphasized that perceiving overeating as a sin helps her eat healthy. Therefore, the role of spirituality in initiating health behaviors among African Americans is not only about perceiving one’s body “as a temple”, but also fearing engagement in “sinful” overindulgence.

Participants also broadly discussed dance and music as factors that are highly appreciated in African American culture and that facilitate healthy lifestyle. The appreciation of dance and movement highlights that exercise does not have to be hard or unenjoyable, it can be a fun activity and a very normal part of one’s life. In describing the importance of dance in her life, *Participant 20* said:

“We all love to dance. I love to dance, where I grew up dance was the exercise you went to; an African weekly dance class or any kind of dance class like we did, all kinds of dance, modern, hip-hop like, even Zumba that used to be my primary way of exercising”.

Reflecting the importance of music and dance in shaping African Americans health, *Participant 13* stated:

“[…] Music. I think one thing you know, we’re trendsetters. That’s, you know, um … if given the opportunity, we’re an active people […] when we don’t have the opportunity, we find a way sometimes to enjoy ourselves and dancing, you know, is a strong culture in the black community”.

Participants indicated that they stay active through non-traditional means of physical activity such as dancing, a cultural activity embedded in the black community. Similarly, *Participant 4* emphasized the importance of dance saying:

“[…] The music, the rhythm, and dance - it is all like a big healthy thing but it’s not framed as that. Like when kids go to dance and you see them dancing, I mean their dances are very aerobic. You don’t always think of it as them exercising but it is”.

#### 3.1.2. Difficulties Changing a Culturally Ingrained Proclivity for ‘Unhealthy’ Behaviors

While discussing cultural factors that influence health-compromising behaviors, the majority of participants focused on culturally preferred, traditional ethnic foods which they described as mostly “unhealthy”. Cooking with fat, such as lard, seasoning heavily with spices and salt, eating pork meat frequently—all these food consumption practices, derived from long standing tradition within the culture, were described as impeding a healthy lifestyle within African American communities. Because these practices are deeply ingrained within the culture, they are perceived as difficult to change. As *Participant 14* explained:

“Within our community we’ve always been like, “the greasier the food, the better”. We still like cooking with lard and, you know, it is just ingrained […] it’s literally a passed down thing from one generation to another”.

Several participants expressed that, although most African Americans realize that some aspects of their traditional cuisine are unhealthy, ethnic food is comforting and allows for connectedness, expression of one’s cultural identity, and the connection to African American culture. As *Participant 13* noted:

“It [traditional ethnic food] is a cultural connotation and it’s also almost a spiritual thing. It’s, you know […] And a loving thing because it’s a part of this joy in cooking, and there’s that, you know, feeling of love you’re preparing for your family”.

However, people reported feeling conflicted between their desire to unite around their ethnic food, and their awareness of the fact that their traditional food may not always be the healthiest. For example, *Participant 8* said:

“Soul food’ is not the healthiest food; and the food people typically choose to eat, as a culture, isn’t the most healthy. But there’s strong family bonds that happen around this food. So it’s kind of a disconnect. Family bonds are strong, but the nutrition typically isn’t that great”.

Noteworthy is how participants indicated that their traditional ethnic food strengthens bonds with their families and their culture, but this type of food is also unhealthy. The conflict surrounding African Americans traditional ethnic foods is evident in *Participant 4*’s words: “it’s a comfort food because we’ve united around that but it’s not healthy food. And how do you change that without feeling like you’re somehow stepping away from your culture”?

#### 3.1.3. The ‘Healthy’ Individual: Aligning Health Beliefs and Behaviors with that of Mainstream Culture

In describing healthy aspects of their mainstream culture, many participants indicated that the recent trend of promoting healthy lifestyle within their mainstream culture seems to be an important factor encouraging their healthy behaviors. Quite frequently participants mentioned that mainstream culture promotes “adapting more vegetarian lifestyle” (Participant 4), “controlling one’s portion size” (*Participant 3*), “learning about healthy options” (*Participant 9*), and “eating in moderation” (*Participant 19*). According to participants, healthy lifestyle was more frequently promoted within their mainstream American culture than in their African American culture. *Participant 1* expressed this observation by saying:

“I think there’s a lot of values that Americans place on health that are really important; like it’s more of a priority than in African American culture […] I think health should be a priority to people”.

Similarly, when asked what African Americans can learn from their mainstream culture that would help them lead healthy lifestyle, *Participant 16* pointed to a focus on taking care of one’s body, physical health, and wellbeing, saying:

“I feel like we can definitely learn a lot more about just caring for our bodies in general. Even just things like drinking more water, which some of us don’t like to do haha. You know drinking more water and just taking care of like both emotionally and mentally and also physically our well-being. So for sure”.

This sentiment of equating some aspects of mainstream American culture with a strong emphasis on health was echoed in *Participant 6’s* account. She noted that “mainstream America seems to be more focused on health and mental health as well and wellbeing”.

Another important salutogenic property of mainstream American culture that many participants focused on was the importance of being physically active. Participants commented that regular physical activity is promoted within their mainstream culture, and that being active may exemplify itself in many different forms. As *Participant 6* noted:

“Mainstream American culture promotes that you exercise every day; and promotes staying active and keep moving; and even if today’s not basketball today, still go outside and take a walk kind of a thing”.

#### 3.1.4. “The Best of Both Worlds”: Combining Cultural and Mainstream Approaches to Health

Participants were directly asked to share their perspective on the possibility of *picking and choosing* typical healthy behaviors from both cultures in order to optimize their health. They were very receptive to the idea of *picking and choosing* and they had a lot to say about an issue. *Participant 19* described the benefits of this approach by stating:

“Everything that’s out there when it comes to healthy behaviors we can implement into our everyday lives and it could be the best of both worlds; it’s something new and something refreshing and then it’s been a part of you for generations; and putting them both together can be amazing”.

The majority of participants indicated that they indeed incorporate different elements of their ethnic and their mainstream culture into their healthy lifestyle related practices; and that “it’s important to integrate both cultures as there are aspects from each culture that is positive that we [African Americans] can all learn from” (*Participant 20*). *Participant 6*, for example, described her experience in taking advantage of being part of both her ethnic and mainstream cultures to improve her health:

“I *pick and choose*. Okay let’s go to food. So the first one would be not eating out too much, that’s something I grew up with in my ethnic culture, try and cook your own food. But then one thing I adapted from mainstream culture is - like I said, I’m a vegetarian- eating more greens and stuff like that. My answer would be *pick and choose* because I choose what’s good from my culture and I choose what’s good in my mainstream America, and I try to combine it”.

Illustrated in the quote above is the flexibility of belonging to both ethnic and mainstream cultures. People are not bound by African American traditions, and they can move between their mainstream and ethnic cultures. Taking advantage of these two cultures to which African Americans belong further highlights that African Americans are “agentic” (*Participant 20*), actively making decisions about their health.

*Participant 14* echoed the opinion expressed above by stating that *picking and choosing* is a good way “to try new and different healthy things,” “to embrace both ethnic and mainstream culture,” “to inspire other people to lead healthy lifestyle,” and “to avoid boredom”. As she said: “I think it’s best to be able to *pick and choose* things [healthy behaviors] from both cultures so you don’t get bored doing the same repetitive things that you’ve been doing”.

Although people mostly talked about *picking and choosing* within one specific domain (i.e., food or physical activity), sometimes *picking and choosing* healthy behaviors from both cultures involved the mix of food intake and physical activity. This selective choice of the best of both worlds seems to be apparent in *Participant 23’s* words:

“I would say that in terms of eating habits eating more fresh vegetables and fruits and stuff like that, it’s probably more from mainstream American culture, and then more of a focus on athleticism as a woman comes from my African American culture”.

Most participants agreed that *picking and choosing* can be or is (for those who already *pick and choose*) beneficial for their health as they believed that in order to acquire an optimum health, it is essential to incorporate health behaviors from different cultures to which they belong.

Preparing *healthy makeovers* of some of the unhealthy dishes was another commonly identified strategy to optimize one’s health. *Participant 19* emphasized the importance of preparing *healthy makeovers* of some of the less healthy foods saying:

“You can choose to, prepare your traditional meals but opt for a better healthier version. Um don’t heavily season it, don’t overcook it, or um implement different areas of this healthy kick into the foods you’ve loved since you were a kid, and try new things and see what works for you”.

Consuming *healthy makeovers* of some of the less healthy foods without the necessity to give up one’s favorite dishes was described as a valuable, and not overly complicated way to improve one’s health. Some participants indicated that they can still, as *Participant 23* stated, “create foods that […] are staples of African-American diet,” but prepare them in a “healthier way”. *Participant 13* described his experiences with *healthy makeovers* saying:

“I’m going to have my chicken baked instead of fried, how is that? Maybe I’ll have a baked potato instead of French fries. Small things like that. But they [people in the community] need to see that and they need to see other black folks doing it. So it’s, it’s a communal thing and it needs to be in their face”.

Evident in the above extract is the notion that preparing healthy makeover is a gradual process that involves small steps and engages the entire community so that it becomes a “communal” phenomenon.

#### 3.1.5. The Importance of Support from the Ethnic and Mainstream Community

When it comes to lack of social support and aversive social environment, the experiences of discrimination and distrust toward mainstream culture were two of the most commonly mentioned factors rooted in mainstream culture that negatively affect African Americans’ health behaviors and wellbeing. In particular, many people spoke about current racial tension in the United States and their discomfort with regard to “anything mainstream,” and about their vanishing faith in mainstream culture. In describing her thoughts associated with barriers to healthy lifestyle that hail from mainstream culture, *Participant 4* said:

“With everything that’s going on now, you see that well-being, your emotional and physical well-being is very much influenced by the way that you’re treated within these urban environments. And I think there’s a higher percentage of African Americans who are not just, not well, because we know that we kinda live in an anti-black society”.

Similarly, when asked about the factors that compromise African Americans’ health behaviors and wellbeing, *Participant 24* spoke about daily experiences of racism among many African Americans:

“Let’s start with systematic racism. I think it’s different for every person. I was fortunate enough to be able to attend Colombia University so I have slipped through the cracks of oppression and been able to have this great education here. But not everyone can make it out and not everyone can have those opportunities to see past a bunch of negativity whether it be from racist employers or racist police or people who would judge you by your appearance and not know what’s in your head. Um, so I think that’s one thing that’s a daily challenge to a lot of people”.

This point of view was common among other participants. Some people gave a quite vivid and emotional account of the experiences of racism and discrimination that were deemed to be an unfortunate part of many people’s lives and that prevent them from being healthy. Some participants, such as *Participant 4*, spoke about lack of support from mainstream culture of African Americans’ health in general and described the notion that African Americans are seen as lesser within the wider mainstream culture:

“I don’t know, I have very little faith in mainstream America right now and I have very little faith that they care enough to do anything different for the African American community. I think, they would have to see us as human before they would even be able to help us be healthier and I just don’t think that enough of them believe in our humanity”.

Noteworthy is the sense of helplessness in the above account and the degree of dependency on mainstream America to assist African Americans to become healthier.

Just as negative social environment can be detrimental for African Americans’ health, strong social support can enhance individuals’ health behaviors. Evident in participants’ responses is that their social environment, specifically their family and friends, can constitute a powerful catalyst for healthy behaviors. Some participants reported that their loved ones, family and friends often inspired them to eat healthy and exercise. For example, *Participant 5* commented on the influence of people’s social environment on their engagement of health behaviors by saying that:

“Having friends who do it [lead healthy lifestyle] can help – if you know that group of people [that] work out and run from Central Park and back, I feel like that you’re more likely like to engage in that type of behavior if you know your friends are doing it”.

Furthermore, *Participant 16* suggested friends often provide a necessary support that prevents people from indulging in unhealthy food, stating:

“My friend and I really encouraged each other. So I would be like “oh I’m really dying for chicken wings” she would be like “No don’t do it!”. And so like having that support that is really great. She would be like “oh I was at work today and I’m thinking about having a donut” and I’m like “don’t do it! You don’t want to do it”!

Some participants mentioned the role of family in highlighting different concerns related to health, such as delayed financial burden that can be triggered by unhealthy lifestyle choices, or the preference for a lean body type. Delayed financial burden refers to future financial consequences of current unhealthy practices. For example, *Participant 2* stated:

“One of the things that my family really pressures me to do this eat healthy now because of the financial burden that it brings on later. So like my dad who has high blood pressure and diabetes […] and he has to pay about $900 a month for medicine because of the choices that he made earlier in life so he encourages me to eat healthy and have a healthy lifestyle because of the financial burden it’s caused. My mom on the other hand, she favors having a small frame being lean, being skinny and she pressures me to eat healthy because of that”.

Other family members may provide indirect motivation for one to stay healthy. Reflecting on the role of family in her health, *Participant 19* said:

“Family members with high blood pressure, and, some family members with diabetes motivate me a little bit more to want to continue exercising and healthy eating just so I won’t have those complications later on in life”.

This suggests that pursuing healthy lifestyle African Americans may not only be inspired by healthy behaviors of their family members and friends, but they also can be influenced by fear that comes from their willingness to avoid poor health in the future.

Additionally, participants often talked about bi-directional, cross-generational influence of family members on one’s health. It seems as though both older and younger members of one’s family can contribute to the improvement of other family members’ health. For example, *Participant 10* reflects on these bi-directional influences between younger and older generation and on the importance of positive modelling behavior saying:

“My mum makes smoothies, she’s tried juicing […] And like she goes to the gym sometimes, but mostly if I’m with her, she’s more motivated to go. So I guess that companionship helps her to be more inclined to work out […] I think I do influence my family too. ‘Cause I’m always talking about eating healthy and you know they joke around with me about like, oh you’re eating this and it’s not gluten free, like so they’ll poke fun at me but I definitely influence them. I feel like they have made some improvement in their food options as far as leaning more toward the healthy side because of me, so….”

While it is important to understand African Americans’ mainstream and ethnic cultures in the context of the facilitators of and barriers to healthy food intake and physical activity, it is equally important to examine factors that directly affect the influence of culture on individuals’ health. In other words the ability of culture to influence people’s health depends on other practical considerations beyond culture.

### 3.2. Practical Considerations beyond Culture When Adopting a Healthier Lifestyle

#### 3.2.1. “It’s is not worth it to eat healthy”: The Cost of Having a Healthy Lifestyle

Most participants reported that being able to afford fresh fruits and vegetables would make it much easier for them to engage in a range of different health behaviors that are typical in both ethnic and mainstream cultures. Most participants indicated that “income” influences “what and how healthy” people eat within African American communities. Lack of affordable food and exercise options was one of the major barriers to healthy living for African Americans. Therefore, providing affordable options with regard to healthy eating and exercising was identified as one of the most important factors that can facilitate healthy food intake and physical activity, as *Participant 16* said:

“I think having things that are affordable. That’s number one. That’s a pretty big issue. If it’s something that’s free in the neighborhood or it’s more affordable for them [African Americans] I feel like that will make them more like “okay I can do this. I can afford $10 once a week”. So I definitely feel like having more affordable options for fitness and for food is definitely would help us”.

Several, participants spoke about “the cost-benefit analysis” that they engage in daily when making decisions about the type of food they purchase, explaining that they are often faced with a dilemma to either pay more and get smaller amount of healthy food or pay less and get larger amount of unhealthy food. *Participant 10* described this dilemma saying:

“If you have a choice between an apple for $1 for a full meal for $1, that may not be the healthy choice but that’s gonna make you full, you will choose the second option”.

Similarly, *Participant 18* said: “Why go buy a tomato for four dollars when I can go get four burgers at McDonald’s for four dollars too”?

In general, participants expressed the belief that, in order for African Americans to engage in healthy behaviors from both their ethnic and mainstream culture, the healthy choices need to be more affordable. That is, there needs to be economically reasonable alternatives to the unhealthy food options that are both easily accessible and affordable. This belief is exemplified by the following statement from *Participant 24*:

“I think that people would consider eating healthy it if it was economically feasible, but you know if you have cheaper ingredients, if it’s cheaper to use butter than it is to use olive oil, then they’re going to use the butter”.

This notion of “it is not worth it to eat healthy” was common among participants and was described as one of the reasons for poor health behaviors within their communities.

#### 3.2.2. Access to Healthy Foods, Healthy Living, and Education

Several participants indicated that it is essential for African Americans to be able to afford healthy foods, but these options need to be readily available to them. Consistent with this notion, *Participant 15* stated:

“If healthy food and wellness, and lifestyle was sitting right there in front of you, I don’t think anybody’s choosing unhealthy option. It’s not like if presented with option A – a life of prosperity, happiness, wellness, healthy, long-life, longevity, and option B – devastation, terrible diet, health, diabetes, anyone would say “I think I want B”, you know. So if it’s not presented to you don’t even know you can choose it”.

Some participants explained that having access and exposure to healthy food can make a difference in many African American communities. This notion is exemplified by the following excerpt from *Participant 14*:

“If we have the access … to fruit stands and the stores and the, you know, the health foods stores that they tend to have in other areas, I think it would work; I just think that we have to actually be able to see it in the area, you know. ‘Cause if we see it we’re gonna to be intrigued by something new. So I think if you put it within the community I think it’ll work because initially we do wanna live a long period of time I think like everyone else”.

Another frequently identified factors that can help African Americans enhance their health behaviors was health education. Knowing what is considered healthy, how to prepare healthy meals, and where to find an affordable place to exercise were identified by participants as common concerns. For example, *Participant 16* summarized the importance of education in shaping community’s health saying:

“Education I feel like is number one. So for people to know exactly where to find healthier options for food, where to find your exercise programs or fitness programs within their community so you don’t have to travel so far”.

*Participant 4* shared a similar thought:

“Showing people how to incorporate different foods into your diets is important. And now I’m ordering cookbooks and stuff so I can see different foods and spices, but like for people who haven’t ventured out to learn about different healthy foods, they don’t know what they eat, or how food affects their body, or how to cook them. So education is a key”.

In general, participants often discussed education in the context of awareness of what constitutes healthy lifestyle and in which creative ways African Americans can learn about healthy practices.

#### 3.2.3. Personal Factors and Motivation

Many participants described personal motivation as an essential ingredient in the pursuit and maintenance of healthy lifestyle. For example, Participant 16 expressed his opinion about the role of motivation in pursuing healthy behaviors, saying:

“I feel like it [healthy lifestyle] has to first start with you motivating yourself. Some people feel like maybe it’s too late, I’ve gotten too far, I can’t start now. So that self-doubt probably is already there”.

Similarly, with physical activity, many people identified motivation as a factor that “helps them start and then keeps them going”. Personal motivation and commitment to a healthy lifestyle were indicated as essential components of taking advantage of healthy behaviors people are exposed to in both their ethnic and mainstream cultures. As *Participant 8* said, “If someone is not motivated, it’s hard to have them change […] I think that’s the biggest thing, you know, prioritizing yourself”.

## 4. Discussion

The present study explored: (1) the role of people’s ethnic and mainstream cultures in shaping their health behaviors; (2) how individuals perceive the idea of *picking and choosing* healthy behaviors from their ethnic and mainstream culture to optimize their health; and (3) psycho-socio-environmental factors that optimize the positive influence of culture on African Americans’ food intake and physical activity. The findings illustrate the importance of both ethnic and mainstream cultures in African Americans’ food intake [11,16] and physical activity [17]. In particular, the present study found that African Americans position both their ethnic and mainstream culture as important influences on their health behaviors. Thus, the present findings extend previous research by highlighting the simultaneous influences of both African Americans’ ethnic culture and mainstream American culture on their food intake and physical activity and by emphasizing the importance of taking the advantage of “the best of both worlds” (that is, combining cultural and mainstream approaches to health).

With respect to their ethnic culture, and consistent with previous research, participants often mentioned resilience [33], reliance on spirituality [11,42], dance and music [43] and social support [28] as important health-enhancing qualities endorsed in their African American culture. In contrast, traditional “soul food” cuisine was reported as one of the most important cultural factors that compromises healthy food intake within African American communities [43]. This finding may explain why preparing *healthy makeovers* of some of the less healthy traditional African American dishes appealed to participants in the present study. The importance of *healthy makeovers* identified in this study is consistent with previous research [11,16] and suggests that African Americans may embrace the idea of preparing healthier versions of some of the unhealthy traditional dishes in order to improve their health yet maintain connect with their cultural traditions.

With respect to individuals’ mainstream culture, participants indicated that “healthy lifestyle trends” popularized within their mainstream culture and the importance placed on physical activity are two the most important salutogenic qualities of their mainstream culture. In contrast, and consistent with previous research [44], the experience of discrimination within one’s mainstream culture was reported as one of the biggest health compromising factor. All these findings suggest that ethnic and mainstream cultural norms and behaviors around food intake and physical activity can be important in shaping African Americans’ health behaviors.

Given that African Americans belong simultaneously to their ethnic and their mainstream cultures, the current study also examined individuals’ reactions to the idea of *picking and choosing*, that is incorporating the healthy elements of both cultures into their lives. Most participants reported engaging in *picking and choosing* healthy behaviors related to food intake and physical activity from both cultures to which they belong. They also perceived *picking and choosing* these behaviors as a way to optimize their health. The potential benefits of incorporating normative behaviors from one’s ethnic and mainstream cultures is consistent with the concept of Bicultural Identity Integration [22], which is associated with greater wellbeing, creativity, problem solving, and coping strategies [23]. In the context of current study, one possible explanation as to why engaging in behaviors from both cultures may be beneficial for African Americans’ health is that doing so provides individuals with a wider range of health behaviors to choose from as opposed to relying on just one source of information about health and wellbeing. Belonging to more than one culture exposes individuals to a broader range of norms, values, and beliefs on the basis of which they can govern their behaviors [13,22], including their health behaviors.

There are also a variety of practical considerations (environmental and personal factors) that participants described as influencing their opportunities to engage in healthy behaviors from both cultures. Consistent with previous research, participants in the current study reported that affordability [45], access to healthy food [30], health knowledge [46] and personal factors [47] are important in determining African American’s health behaviors. The current study, however, builds on previous research by examining these psycho-socio-environmental factors in association with individuals’ ability to incorporate healthy behaviors from both their ethnic and mainstream cultures (i.e., to *pick and choose* and to *consume healthy makeovers* of unhealthy dishes). For example, the willingness to consume fruits and vegetables, which are heavily promoted within mainstream culture, depends on a proper access to these types of foods. This finding suggests that the influence of ethnic and mainstream culture on African American’s health behaviors should be examined in the relation to their broader psycho-socio-environmental context (consistent with ecological models of health behaviors, such as the PEN-3 model [10]).

The results of the present study may have some implications for planning and designing health promotion programs for African Americans. First, the fact that participants show favorable attitudes toward *picking and choosing* and toward *healthy makeovers* suggests that they are open to taking advantage of healthy behaviors promoted in both their ethnic and mainstream cultures. Although there is the potential for both positive and negative influence of ethnic and mainstream cultures on health, these findings further suggest that health interventions could focus on positive health enhancing behaviors already celebrated within individuals’ cultures [12], rather than focusing solely on eliminating health-compromising behaviors. Shifting from a deficit model to a strength-based model empowers ethnic minority groups and validates their experiences without unnecessarily pathologizing their health practices [16]. Of course, research is needed to determine the extent to which incorporating practices from both cultures (including *picking and choosing* and *consuming healthy makeovers*) is an effective means of improving the health and wellbeing of African Americans. Additionally, given that cultural practices and norms can be in conflict with one another and contribute to unhealthy behaviors surrounding food intake and physical [18,19], future studies should simultaneously examine the impact of positive cultural influences, negative cultural influence, and cultural conflicts on individuals’ health behaviors.

Second, by providing an opportunity for individuals to decide which behaviors are important to them, the *picking and choosing* approach resembles the “cultural tailoring” framework of health promotion [14]. This health promotion model is directed toward individuals and cultural subgroups, and acknowledges within-culture differences among people, taking into consideration personal choices and preferences. The autonomy and freedom to make a choice is one of the most fundamental human needs affecting individuals’ performance, relationships, and wellbeing [48,49]. Being able to make a choice increases individuals’ intrinsic motivation and self-efficacy, which further enhance adherence to behavioral change goals [50,51]. Overall, having the opportunity to choose health behaviors that individuals want to pursue is gratifying, desirable, and important from health promotion perspective.

Third, there were a number of psycho-socio-environmental factors that were associated with the likelihood of *picking and choosing*. Targeting these factors in health interventions for African Americans could potentially increase the effectiveness of these interventions. For example, health promotion programs could design interventions that teach individuals how to select or *pick and choose* healthy behaviors that both of their cultures have to offer. Such a self-efficacy building approach to health promotion is empowering, and fosters ownership of individuals’ behaviors, which can further increase the likelihood that they will engage in a variety of healthy behaviors [52].

Finally, the effectiveness of tailoring dietary messages may depend on individuals’ connection to their cultures [53] as well as on differences within the culture [54]. For African Americans, this means that their willingness to *pick and choose* health behaviors from their ethnic and mainstream cultures, and their willingness to *consume healthy makeovers*, could depend on their level of connection to both their ethnic and their mainstream cultures. As Kreuter et al. [54] noted, African American culture does not comprise of a monolithic cultural milieu, and people’s relationship to their culture can vary [54]. Therefore, health promotion programs for African Americans need to consider these variations and individuals’ connections to their culture in the design and delivery of effective health promotion programs. For instance, in order to optimize the effectiveness of such programs, the focus and the content of health intervention can be tailored to individuals’ level of connection to their ethnic and mainstream cultures. Moreover, these programs should also target personal factors such as motivation for leading healthy lifestyle because these factors are potentially important in triggering healthy behaviors.

Although this study revealed some important insights regarding African Americans’ food intake and physical activity, there are some limitations that should be considered. First, the convenience sample consisted of African American students or alumni from Columbia University, or community members predominantly living in the Columbia University area. The sample is, therefore, not representative of the entire African American population. Living in an urban area as opposed to in the rural community can affect African Americans health behaviors differently. For example, these two groups may differ substantially in their access to healthy food and recreational spaces, as well as socio-economic status affecting their health behavior choices. Second, the participants in the study consisted of predominantly educated individuals, and mostly women. Interviews with individuals representing different educational levels and with more men as well might have yielded different conclusions given that both of these characteristics can affect food intake and physical activity. Third, the way participants perceive their African American culture and their mainstream culture may be tied to their level of education and their socioeconomic status, which were relatively high among the research participants in this study. Individuals with higher socioeconomic status might be more connected to their mainstream culture and therefore more willing and able to engage in healthy lifestyle practices common in their mainstream culture. Unfortunately, we did not collect comprehensive demographic and socioeconomic data, which limits our ability to fully understand the sample characteristics and how they relate to participants’ responses. Therefore, future studies should clarify how socioeconomic status affects African Americans’ perceptions of food intake and physical activity common in their ethnic and mainstream cultures. Fourth, most questions designed for this study explored participants’ perceptions of other African Americans’ health behaviors in the context of their ethnic and mainstream cultures; many fewer questions addressed participants’ perceptions of their own behaviors. Because different processes can be involved in perceiving one’s own versus others health behaviors, it would be important for the future research to distinguish these two types of views—of one’s own beliefs and behaviors and those of others. Finally, in terms of our conceptualization of the influence of culture on individuals’ food intake and physical activity, the current study has focused on only two dimensions of this influence (i.e., barriers and facilitators of healthy lifestyle). The rationale for this choice of dimensions is that it reflects the study’s research questions; it has not been examined before in the context of individuals’ ethnic and mainstream cultures; and it has implications for health promotions programs. However, there are many other ways in which the influence of African Americans’ culture on their food intake and physical activity could be conceptualized, and future research would benefit from exploring those dimensions. Despite these limitations, the present research contributes to broadening the understanding of how African Americans make sense of their own experiences in relation to food intake and physical activity.

## 5. Conclusions

Overall, the findings of the present study demonstrate the importance of both ethnic and mainstream cultures in shaping African Americans’ health behaviors. These influences need to be understood in the context of other psycho-socio-environmental factors that affect individuals’ health behaviors. Given that most minority groups are often disproportionately affected by overweight and obesity, and by poor health in general [1,2,4], it is important to examine ways in which people can optimize their health by taking advantage of the diverse values, beliefs, and practices that exist in their ethnic and mainstream cultures. Healthy food intake and physical activity also need to be considered and understood in the context of one’s larger social and physical environment. These findings have implications for health promotion programs directed at African Americans.

## Figures and Tables

**Table 1:** Demographics of the Sample in the Study.

Demographic Characteristics	*n*
Gender	
Female	20
Male	5
Education	
Completed high school	4
BA	2
MA	17
PhD	2
Cultural Identification, self-identified	
Identified more with their ethnic culture	6
Identified more with their mainstream culture	1
Identified equally with both cultures	14
Other	4

**Table 2:** Interview Script, Questions, and Probes.

**Please think for a moment about the values, strengths, and beliefs that are common in your African American culture. Also think about the behaviors that are common in your African American culture that relate to health and wellbeing (defined as the state of being comfortable, healthy, and happy). Now I want to ask you some questions related to the connection of these values and strengths to healthy lifestyle and wellbeing.**	
1	In general, what do you think helps African Americans living in urban areas: (1) eat healthy; (2) be active; and (3) increase their wellbeing? (Probe: please think about specific behaviors, e.g., ways of preparing food, portion size, food labels, type of exercise etc.)
2	In general, what do you think makes it difficult for African Americans living in urban areas to: (1) eat healthy; (2) be active; and (3) increase their wellbeing? (Probe: please think about specific behaviors, e.g., ways of preparing food, portion size, food labels, type of exercise etc.)
3	Which cultural strengths, beliefs, or values do you think can help African Americans living in urban areas: (1) eat healthy; (2) be active; and (3) increase their wellbeing?
4	What can mainstream American culture learn from African Americans about healthy lifestyle, specifically regarding: (1) eating healthy; (2) being active; and (3) wellbeing? (Probe: Any specific behaviors you may think of?)
5	What can African Americans learn from mainstream American culture about healthy lifestyle specifically related to: (1) eating healthy; (2) being active; and (3) wellbeing? (Probe: Any specific behaviors you may think of?)
6	As someone living in the US, you are exposed to components of both your African American culture and to mainstream American culture. When you think about the healthy lifestyle behaviors that you engage in, would you say that these are more common in mainstream American culture, or more common in your African American culture? Do you *pick and choose*, so to speak, different health behaviors from both cultures?
7	Do you think that African Americans can *pick and choose* healthy behaviors and practices from both their mainstream and ethnic culture in order to optimize their health? Can they do so without compromising their cultural identity? (Probe: Would it be difficult? Or easy? Do you do it? Do you know people who do it? What’s difficult about it, if anything? What is easy about it, if anything? What would make it easier for people to do it?)
8	What should members of mainstream American culture do more of to facilitate the improvement of health and wellbeing, specifically healthy eating, and exercise, of African American communities? (Probe: Think of health providers, individuals, and mainstream organizations such as schools or work places.)
**Now, I want you to talk specifically about those people in your African American culture who try to lead a healthy lifestyle. Please think about specific person or a group of people you know.**	
1	What do they typically do that helps them lead a healthy lifestyle? (Probe: Any specific behaviors you may think of related to (1) eating healthy; (2) being active; and (3) wellbeing?)
2	What helps them engage in these behaviors? (Probe: The role of other people, family friends?)
3	How does your family affect your healthy lifestyle choices related to: (1) eating healthy; (2) being active; and (3) wellbeing?
4	How do your friends affect your healthy lifestyle choices regarding: (1) eating healthy; (2) being active; and (3) wellbeing?
Is there anything else that you would like to add that could be important to understanding health behaviors and healthy lifestyles for African Americans?	

## Appendix A. Participants’ Level of Education and Cultural Identification

**Table:**

Participants’ Number #	Education	Cultural Identification
Participant #1	4	4
Participant #2	3	1
Participant #3	3	3
Participant #4	4	1
Participant #5	3	3
Participant #6	3	3
Participant #7	3	1
Participant #8	3	3
Participant #9	3	4
Participant #10	3	1
Participant #11	2	3
Participant #12	1	3
Participant #13	3	3
Participant #14	3	1
Participant #15	3	4
Participant #16	3	3
Participant #17	3	3
Participant #18	1	1
Participant #19	1	3
Participant #20	3	3
Participant #21	3	3
Participant #22	1	2
Participant #23	3	3
Participant #24	3	4
Participant #25	2	3

*Note:* **Education**: 1 = high school, 2 = BA, 3 = MA; 4 = PhD; **Cultural Identification**: 1 = Identified more with ethnic culture, 2 = Identified more with mainstream culture, 3 = Identified equally with both cultures, 4 = Other.
